# Coupled mechanical creep and bio-compression and residual settlement in a multi-stage municipal solid waste landfill, Korea

**DOI:** 10.1038/s41598-022-21872-3

**Published:** 2022-11-09

**Authors:** Jo Young-Seok, Yoo Wan-Kyu, Hwang Sung-Phil, Kim Chang-Yong

**Affiliations:** Korea Institute of Civil Engineering and Building Technology, Goyang, Republic of Korea

**Keywords:** Environmental sciences, Engineering

## Abstract

Based on a field monitored dataset measured at landfill #1 over 21 years, the characteristics of settlement coupling mechanical creep and biodegradation and the residual settlement were analyzed. Since landfill #1 is a multi-stage municipal solid waste (MSW) landfill where dykes are constructed after landfilling for subsequent waste fills, the waste decomposition between the lower and upper lifts was quite different and it is difficult to discern between the mechanical creep and bio-compression on the settlement curves. The compression ratio coupled with mechanical creep and bio-compression and the residual compression ratio were determined as 0.233 and 0.068, respectively. This implies that biodegradation was gradually and significantly reduced in the MSW settlement behavior after the residual settlement began. The starting date of residual settlement was distributed between 3821 and 5402 days from the initial date of landfilling. The settlement coupling mechanical creep and biodegradation (*S*_MB_) was 2.9 times larger than the residual settlement (*S*_RS_), and the duration of *S*_MB_ is determined to be 0.3 times that of *S*_RS_. In addition, the remnant methane gas content existed in the landfill gas, and low-level biodegradation was still generated in the waste buried for more than 10 years after the residual settlement began.

## Introduction

After waste disposal is completed in a landfill, a final cover is laid on its surface, and the space on the landfill surface could be used as public places such as parks, golf courses, and various facilities^1^. However, significant settlements can be generated in completed municipal solid waste (MSW) landfills due to the decomposition of organic matter, i.e., biodegradation^2–5^. This settlement could damage the public facilities or superstructures placed on the landfill surface, and thus they should be constructed after active biodegradation is considerably reduced. Therefore, it is necessary to determine the point of time when the organic matter in the landfill has almost completely disappeared and residual settlement starts.

Geotechnical researchers have studied the settlement phases and mechanisms, i.e., the long-term MSW settlement behavior, to investigate this point for decades. Grisolia and Napoleoni^6^ mentioned that active biodegradation decreased within 11–33 years from the beginning of waste disposal, depending on the waste composition, and Hossain and Gabr^7^ stated that the residual settlement would occur when 3500 days elapsed from the start of waste disposal. Fei and Zekkos^8^ reported that, based on a series of laboratory tests, the biodegradation-dominant settlement was almost finished after 20.0–35.3% of the total duration had elapsed. Kumar et al.^9^ mentioned that the behavior of MSW in landfills is influenced by the complex coupled interactions of different phenomena which mainly include hydraulic, mechanical, and biochemical processes. Although several studies have been reported, research on the residual settlement behavior of MSW landfills based on field-monitored data on long-term waste settlement is rare. This is because it is difficult to obtain data measured for decades at landfill sites for several reasons, e.g., the malfunctioning of settlement plates due to physical and chemical corrosion. It also remains unknown for how long MSW landfills have to be monitored as existing landfill sites are still in aftercare period^10^.

In addition, the waste settlement mechanisms can be expressed as the waste settlement characteristics, i.e., compression index or ratio, and many researchers^2,8,11–32^ reported these values based on the results from laboratory or field tests. Geotechnical engineers have referred to these values to understand the behavior of long-term MSW landfills.

In this study, the long-term waste settlement behavior during post-closure was examined using a field-monitored dataset measured at a multi-stage MSW landfill over 21 years. Based on time-series analysis, the date on which the biodegradation of organic matter was significantly reduced and the residual settlement mechanism became dominant was determined as well as the compression ratios coupling mechanical creep and biodegradation. The residual compression ratios were also calculated and compared to the compression ratios coupling mechanical creep and bio-compression. The waste settlement coupling mechanical creep and biodegradation was compared with the residual settlement and the duration. In addition, the relationship between the long-term waste settlement mechanisms and the history of landfill gas (LFG) collected at a multi-stage MSW landfill was analyzed.

## Materials and methods

### Settlement phases and mechanisms

**Figure 1:**
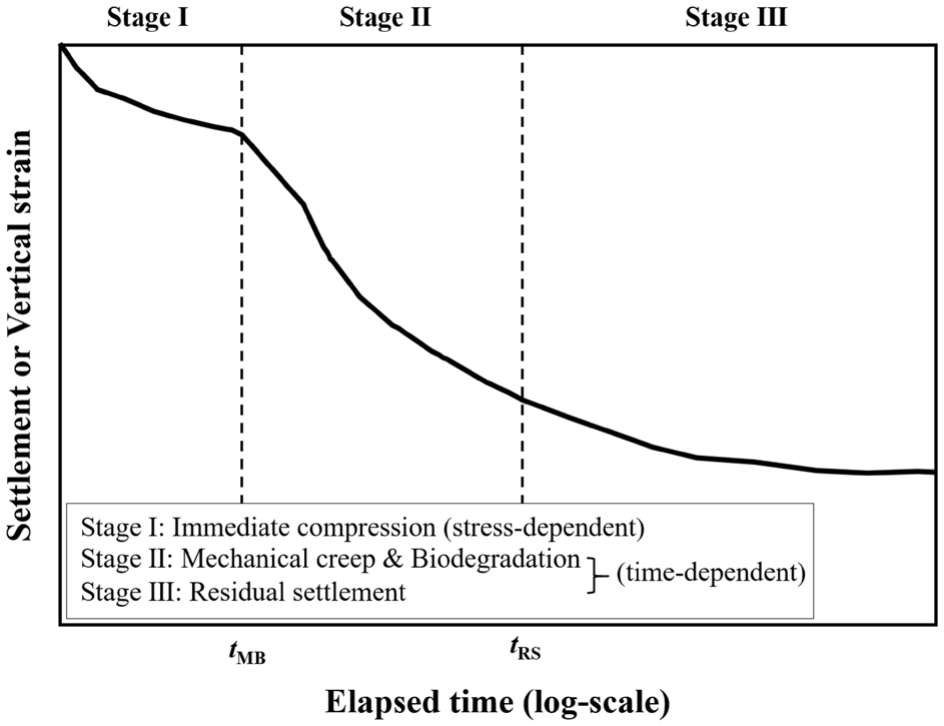
Conceptual waste settlement mechanisms of MSW landfills.

Based on the soil mechanics theory, Sowers^11^ described the stress- and time-dependent settlements (*S*_SD_ and *S*_TD_) as primary and secondary settlements. Bjarngard and Edgers^33^ subdivided the time-dependent settlement into settlement by mechanical creep and the settlement by biodegradation of organic matter in waste. Hossain and Gabr^7^ proposed that the time-dependent settlement could be further subdivided into residual settlement by mechanical creep (*S*_RS_). Chen et al.^34^ recently stated that mechanical creep and biodegradation should be coupled in the waste settlement mechanisms because it is difficult to discern the two settlement behaviors in the settlement curves measured at landfill sites. Based on their statement, the waste settlement mechanisms can be divided into three stages (Eq. (1); Fig. 1).

1 $$S_{T} = S_{SD} + S_{TD} = S_{pri} + S_{MB} + S_{RS}$$

The stress-dependent settlement (Stage I in Fig. 1) primarily occurs by additional weight of waste, loading, and the discharge of gas or water in the voids^35^. After the stress-dependent settlement, the time-dependent settlement dominates the waste settlement mechanism. This settlement continues for months, years, or even decades, depending on the waste composition and landfill design^1,3–5^. In this study, the time-dependent settlements were subdivided into waste settlement coupling mechanical creep and biodegradation (*S*_MB_; Stage II in Fig. 1) and residual settlement (*S*_RS_; Stage III in Fig. 1). Hossain and Gabr^7^ stated that waste settlement by mechanical creep occurs in landfill sites between 0 and 1000 days after the primary settlement is completed. In terms of the settlement by biodegradation, i.e., bio-compression, geotechnical researchers^5,13,19,33,36^ reported that bio-compression is a significantly important factor distinguishing the settlement mechanism of soil and waste, and it is usually reported to be 12–21% of the initial waste height in the sanitary landfills for decades. The residual settlement is the final stage of the waste settlement mechanism. This settlement is caused by the physical mechanisms of waste particles such as physical yielding, reorientation, and raveling^2,7,21^.

### Site condition

**Figure 2:**
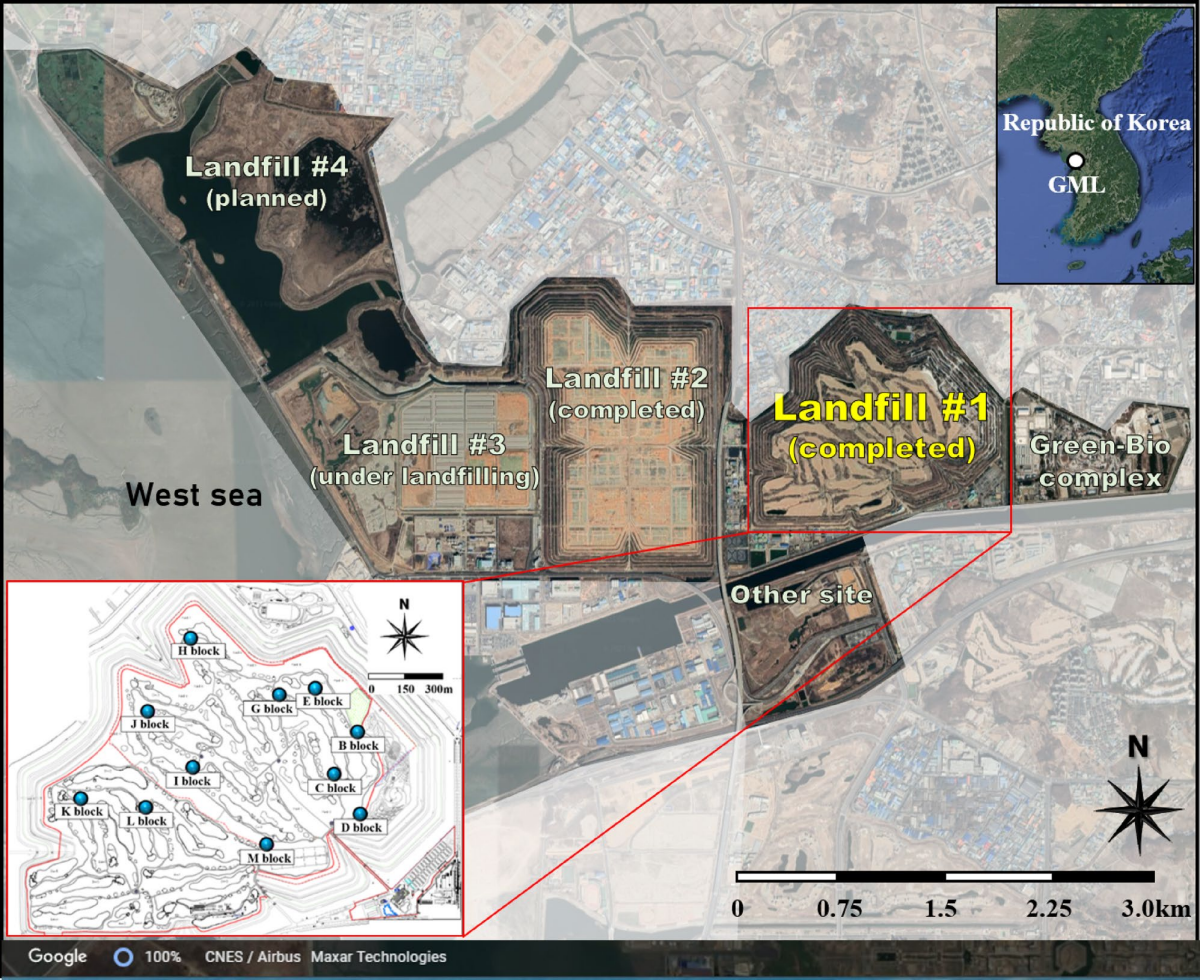
Location of Gimpo Metropolitan Landfill (GML) and landfill #1 where the waste disposal was completed; Layout of the GML and detailed location of each landfill; Location of settlement plates installed on the uppermost layer of landfill #1 after closure (Satellite images were extracted from Google Earth and edited using Microsoft Office's PowerPoint).

The Gimpo Metropolitan Landfill (GML), a multi-stage MSW landfill where dykes are constructed after landfilling for subsequent waste fills, was constructed on soft clay ground with an area of 14.63 km^2^ in Gimpo areas of the Republic of Korea. The GML is one of the biggest landfills in the world. The municipal solid waste (MSW) was buried in the GML since 1992. The GML, composed of four landfills and two complexes (Fig. 2), operates as a sanitary landfill. The thickness of each lift in a landfill was designed to be 5 m, in which the thickness of the waste was 4.5 m and the cover soil was 0.5 m. The initial thickness of each lift varied between 4 and 8 m according to the condition of waste disposal. The area of landfill #1, which was focused in this study, is 4.09 km^2^, and 6.40 × 10^7^ tons of waste were buried over 8 years, i.e., from 1992 to 2000^37^. The landfill duration for each block is listed in Table 1. After landfill #1 was closed, stabilization operations, such as final covers and pipes to collect and transport LFGs, were installed on the uppermost part of the landfill from September 2002 to December 2002. Currently, there is a golf course on the landfill as a public space. The information related to landfill #2–#4, e.g., area, capacity, and landfilling duration, was delineated in Jo and Jang^38^.

**Table 1:** The landfilling duration, the proportion of organic matter, unit weight, and waste height for each block in landfill #1.

Block	Landfilling duration (yyyy.mm.dd)		Organic matter (%)	Unit weight (kN/m^3^)	Waste height (m)
	Start	Completion			
B	1993.01.05	2000.05.26	64.13	10.99	40.01
C	1992.09.25	2000.04.26	63.15	10.99	44.10
D	1993.04.07	2000.05.31	64.16	10.98	40.61
E	1993.06.10	2000.04.02	64.16	10.98	40.14
G	1993.02.26	2000.03.20	64.18	10.99	44.07
H	1994.01.27	2000.04.27	64.24	10.97	39.06
I	1993.03.05	2000.07.01	64.91	10.98	43.60
J	1993.10.23	2000.09.20	64.91	10.98	40.81
K	1993.05.27	2000.09.22	64.95	10.98	42.25
L	1993.01.12	2000.08.11	64.91	10.98	42.11
M	1993.05.10	1999.12.17	64.24	10.98	42.09
Average			64.36	10.98	41.71

The detailed landfilling history for each lift of the blocks is presented in Table S2. The organic matter consists of food, paper, wood, textiles, rubber, and leather (the detailed annual composition is presented in Table S3).

During landfilling, the MSW was buried in the eleven blocks (B, C, D, E, G, H, I, J, K, L, and M) and construction and demolition (C&D) wastes, i.e., sewage sludge, dredge soil, industrial waste, construction waste, and textiles, were buried in the O, P, and Q blocks. In which, the blocks mean the cells as terminology for portions of MSW landfills. The proportions of MSW and C&D are shown in Table S1. The waste disposal in the O, P, and Q blocks was suspended due to the abnormal breaking of the blocks.

The organic matter proportion and unit weight of the MSW buried in the eleven waste blocks (B, C, D, E, G, H, I, J, K, L, and M) and the waste height for each block are listed in Table 1. The organic matter was determined by the following procedures; a) calculated the content of organic matter for each lift based on the waste disposal history in Table S2. In which, the annual organic matter was utilized the value shown in Table S3; b) add up the content of organic matter for each lift; c) the added up value was averaged. The unit weight of each block in Table 1 was also determined by the same method as the organic matter. Jo and Jang^38^ determined the annual unit weight of waste in landfill #1 using the data measured at earth pressure plates. In which, the value presented in Jo and Jang^38^ was utilized in this study. The organic matter consisted of food, paper, wood, textiles, rubber, and leather. The detailed composition of the organic matter in each block is presented in Table S4. The ranges of organic matter and unit weight for each block were 63.15–64.95% and 10.97–10.99 kN/m^3^, respectively. The gravimetric water content of MSW is reported to be 113.7%, which is not an annually reported value. Once the waste disposal was completed, the waste height for each block ranged between 39.06 and 44.10 m (41.71 m on average) (Table 1).

### Waste settlements post-closure

**Figure 3:**
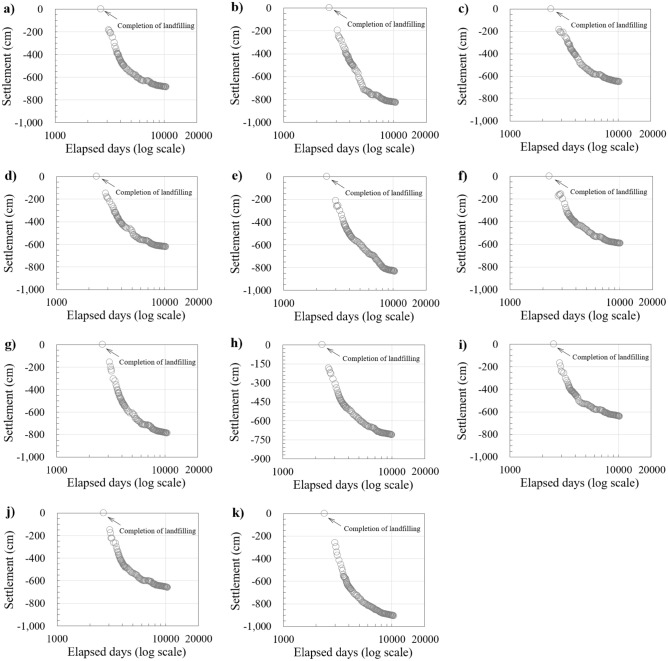
The waste settlement curves measured at each block of landfill #1 over 21 years after closure; (**a**) B block; (**b**) C block; (**c**) D block; (**d**) E block; (**e**) G block; (**f**) H block; (**g**) I block; (**h**) J block; (**i**) K block; (**j**) L block; (**k**) M block.

After the landfill was closed, from December 1999 (M block) to September 2000 (K block), new settlement plates were placed on the surfaces of eleven blocks (B, C, D, E, G, H, I, J, K, L, and M; Fig. 2). There is no settlement plate on O, P, and Q blocks because the landfilling was suspended owing to the abnormal breaking of blocks. The long-term waste settlements of the eleven blocks were measured continuously at the new settlement plates, and this study utilized data collected over 21 years, i.e., from post-closure to December 2021. During this period, the cumulative waste settlements measured from the settlement plates ranged between 5.91 and 9.06 m which corresponded to the cumulative vertical strains ranging from 15.15 to 21.54% (17.21% on average). Based on the content of organic matter buried in landfill #1 (Table S4), the average value is 64.35%. Since about 64% of the total landfill volume has potential waste settlement possibility, the additional waste settlement will be considered for each block. The waste settlement curves of the B, C, D, E, G, H, I, J, K, L, and M blocks show some common trends, with steep slopes initially, which then decrease gradually and form specific inflection points in between (see Fig. 3).

## Results and discussion

### Determination of $C_{{{\text{MB}}}}^{{\prime }}$ and ***t***_RS_ based on time-series analysis

Since the MSW was buried in landfill #1 over a long-term period, the old and fresh wastes varied from the lower to upper lifts. In landfill #1, the biodegradation of old waste in the lower lifts was significantly completed, and that of fresh wastes in the upper lifts was active. Because MSW decomposition was different for each lift, it was difficult to discern between the mechanical creep and bio-compression on waste settlement curves measured after closure (see Fig. 3). Therefore, in this study, mechanical creep and bio-compression were coupled, and the waste settlement characteristics, i.e., compression ratio, were expressed as *C*′_MB_.

**Figure 4:**
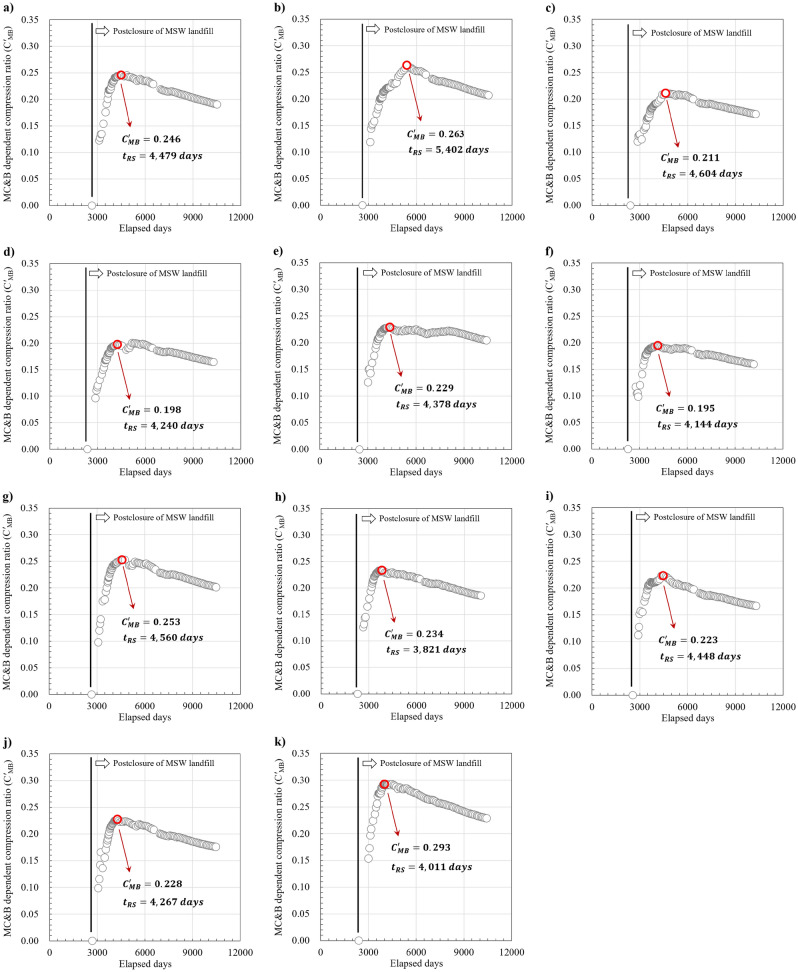
Changes in the compression ratio (*C′*_MB_) coupling mechanical creep and bio-compression to time history, and determination of the starting date (*t*_*RS*_) for residual settlement: (**a**) B block; (**b**) C block; (**c**) D block; (**d**) E block; (**e**) G block; (**f**) H block; (**g**) I block; (**h**) J block; (**i**) K block; (**j**) L block; (**k**) M block.

The coupled compression ratio with mechanical creep and bio-compression (*C*′_MB_) was calculated using Eq. (2), and the time history of *C*′_MB_ is shown in Fig. 4. This equation was modified based on settlement models which were proposed by Sowers^11^ and Bjargard and Edger^33^. In addition, the starting date of waste settlement by mechanical creep and biodegradation (*t*_MB_) was determined as the number of days elapsed from the mid-point time of landfilling duration because landfill #1 was multi-stage landfill and the age of waste between the lower and upper lifts varied. The compression ratio increased after landfill #1 was closed because biodegradation in fresh waste was active and the bio-compression was the dominant MSW settlement mechanism. The compression ratio decreased after the peak value was obtained. In this study, the peak value was determined to be *C*′_MB_, and the elapsed day with the peak value was determined to be the date (*t*_RS_) when the reduction of biodegradation began and the residual settlement mechanism became dominant. The *C*′_MB_ ranged from 0.195 to 0.293 (0.233 on average). The *t*_*RS*_ was distributed in the range of 3821 to 5402 days (4396 days on average). These values were calculated based on the initial date of landfill. The *t*_*RS*_ and *C*′_MB_ for each block are listed in Table 2.

2 $$C_{MB}^{{\prime }} = \frac{{S_{MB} }}{{H \times log\left( {\frac{{t_{RS} }}{{t_{MB} }}} \right)}}$$

**Table 2:** The starting date for the residual settlement (*t*_RS_), compression ratio coupling mechanical creep and bio-compression (*C′*_*MB*_), compression ratio of residual settlement (*C′*_*RS*_) for each block in landfill #1, waste settlement coupling mechanical creep and biodegradation (*S*_MB_), and residual settlement (*S*_RS_) of MSW in landfill #1 measured after the landfill was closed.

Block	B	C	D	E	G	H	I	J	K	L	M	Mean
***t***_**RS**_ (days)	40479	5402	4604	4240	4378	4144	4560	3821	4448	4267	4011	4396
$C_{{{\text{MB}}}}^{{\prime }}$	0.246	0.263	0.211	0.198	0.229	0.195	0.253	0.234	0.223	0.228	0.293	0.234
$C_{{{\text{RS}}}}^{{\prime }}$	0.061	0.047	0.058	0.066	0.103	0.059	0.075	0.074	0.048	0.065	0.090	0.068
*S*_*MB*_ (cm)	521.3 (1833)	720.8 (2974)	526.6 (2584)	459.2 (2439)	507.0 (1533)	417.9 (1776)	589.1 (1895)	508.1 (1716)	458.4 (1543)	498.8 (1855)	669.1 (1805)	534.2 (1996)
*S*_*RS*_ (cm)	164.7 (6031)	105.8 (4920)	124.1 (5275)	162.5 (5480)	325.6 (6399)	173.9 (6118)	198.2 (5934)	203.1 (6033)	182.2 (6206)	161.3 (5935)	237.5 (6222)	185.4 (5868)

The *t*_*RS*_ is the date was counted from the time the landfilling was initiated, and the values in parentheses are the durations (days) of each settlement after the landfill was closed.

### Determination of $C_{{{\text{RS}}}}^{{\prime }}$

The residual compression ratio ($C_{{{\text{RS}}}}^{{\prime }}$) was calculated using Eq. (3) based on the field-monitored long-term waste settlement data. This equation was suggested by Hossain and Gabr^7^ based on the theory of soil-mechanics. These ratios ranged from $C_{{{\text{RS}}}}^{{\prime }}$  = 0.047 to $C_{{{\text{RS}}}}^{{\prime }}$  = 0.103. The average value of $C_{{{\text{RS}}}}^{{\prime }}$ was 0.068 (Table 2). The average of $C_{{{\text{RS}}}}^{{\prime }}$ was approximately 0.29 times the average of $C_{{{\text{MB}}}}^{{\prime }}$. This implies that biodegradation was significantly reduced in the MSW settlement behavior during this period.

3 $$C_{RS}^{{\prime }} = \frac{{S_{RS} }}{{H \times log\left( {\frac{t}{{t_{RS} }}} \right)}}$$

### Comparison between waste settlement coupling mechanical creep and biodegradation and residual settlement

The waste settlement after the landfill was closed was divided into settlement coupling mechanical creep and biodegradation (*S*_MB_) and residual settlement (*S*_RS_). The settlements determined in landfill #1 are listed in Table 2. The *S*_MB_ and *S*_RS_ ranged from 417.9 to 720.8 cm and from 105.8 to 325.6 cm, respectively. The average values of *S*_MB_ and *S*_RS_ are 534.2 cm and 185.4 cm with durations of 1996 days ($\approx$ 5.5 years) and 5868 days ($\approx$ 16.1 years), respectively. The average of *S*_MB_ is 2.9 times larger than that of *S*_RS_, and the duration of *S*_MB_ is 0.3 times that of *S*_RS_.

### Relationship between landfill gas and settlement mechanism

**Figure 5:**
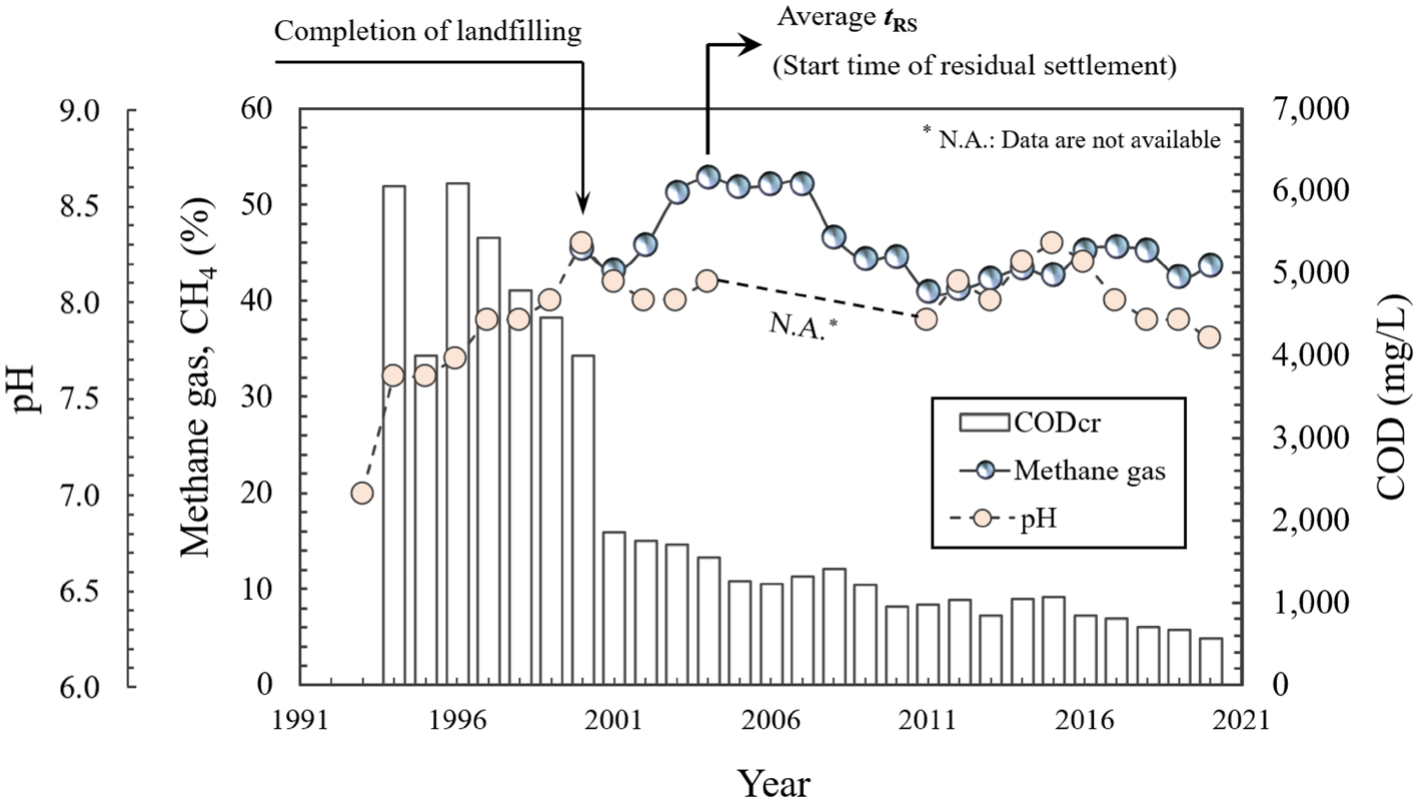
Chemical oxygen demand (COD), methane (CH_4_) gas, and pH of landfill #1 (GLC, 2021).

In landfill #1, the chemical oxygen demand (COD) and pH of the leachate were measured after the landfilling began. The methane (CH_4_) gas was collected since 2000 because the gas collection systems were installed after the landfill was closed. The variations in CH_4_ gas, and COD and pH of the leachate measured at landfill #1 are shown in Fig. 5. The CH_4_ gas increased 3 years after the landfill closure and started to decrease after the peak value in 2004, which corresponds to *t*_RS_, the start time of residual settlement. It implies that there was remnant CH_4_ content in the LFG and low-level biodegradation was still generated in the waste buried for more than 10 years after the residual settlement began. In addition, after the *t*_RS_, the slope of waste settlement curves gradually decreased (Fig. 3) and the compression ratio coupling mechanical creep and bio-compression ($C_{{{\text{MB}}}}^{{\prime }}$) also decreased (Fig. 4). It means that the waste settlement mechanisms have transitioned from the biocompression stage that the biodegradation of organic material is dominant (StageII) to the residual settlement stage that final mechanical creep is dominant (Stage III) as the content of CH_4_ gas was reduced in LFG.

## Conclusions

In this study, the long-term waste settlement of a multi-stage MSW landfill was measured over 21 years after the closure of a landfill, and a field-monitored dataset was investigated. The waste characteristics and settlement behaviors were analyzed using time-series analysis. The following conclusions were drawn from the results:

Since the wastes were buried from the lower to upper lifts at different times over 8 years, it was difficult to discern the mechanical creep and bio-compression on the waste settlement curves measured during the postclosure of the landfill.

The *t*_RS_ and $C_{{{\text{MB}}}}^{{\prime }}$ were calculated through time-series analysis. The $C_{{{\text{MB}}}}^{{\prime }}$ increased after landfill #1 was closed because bio-compression was still dominant in the MSW settlement mechanisms. The trend of $C_{{{\text{MB}}}}^{{\prime }}$ decreased after the peak value was obtained, and this peak value was determined to be the $C_{{{\text{MB}}}}^{{\prime }}$ for each block in landfill #1. The elapsed day with the peak value was determined to be the *t*_RS_. The *C*′_MB_ ranged from 0.195 to 0.293 (0.233 on average). The *t*_*RS*_ was distributed in the range of 3821 to 5402 days (4396 days on average).

The $C_{{{\text{RS}}}}^{{\prime }}$ was also calculated, and it ranged from $C_{{{\text{RS}}}}^{{\prime }}$ = 0.047 to $C_{{{\text{RS}}}}^{{\prime }}$ = 0.103. The average value of $C_{{{\text{RS}}}}^{{\prime }}$ was 0.068. The average of $C_{{{\text{RS}}}}^{{\prime }}$ was approximately 0.29 times the average of $C_{{{\text{MB}}}}^{{\prime }}$. This implies that biodegradation was significantly reduced in the MSW settlement behavior during this period. In addition, the *S*_MB_ was 2.9 times larger than the *S*_RS_. The duration of *S*_MB_ was determined to be 0.3 times that of *S*_RS_.

The landfill gas (LFG), especially methane (CH_4_) gas, was still generated after the *t*_RS_. This implies that remnant CH_4_ content existed in the LFG and low-level biodegradation still occurred in the waste buried for more than 10 years after the residual settlement began. In addition, after the *t*_RS_, the slope of waste settlement curves gradually decreased and $C_{{{\text{MB}}}}^{{\prime }}$ also decreased. It means that the waste settlement mechanisms were transitioned from the biocompression stage that the biodegradation of organic material is dominant (Stage II) to the residual settlement stage that final mechanical creep is dominant (Stage III) as the content of CH_4_ gas was reduced in LFG.

The findings of this study could help to decide the appropriate construction time for public facilities on the closed landfill sites after the residual settlement is initiated. We also expect that the compression ratios, i.e., $C_{{{\text{MB}}}}^{{\prime }}$ and $C_{{{\text{RS}}}}^{{\prime }}$, can be appropriately used for evaluating parameters of other waste settlement models.

## Supplementary Information

Supplementary Information.

## Data Availability

All data generated or analyzed during this study are included in this manuscript [and its supplementary information fles].

## List of symbols

*S*_T_: Total settlement
*S*_SD_: Stress-dependent settlement (= *S*_pri_)
*S*_TD_: Time-dependent settlement (= *S*_MB_ + *S*_RS_)
*S*_pri_: Primary settlement (= immediate settlement)
*S*_MB_: Waste settlement coupling mechanical creep and biodegradation
*S*_RS_: Residual settlement by mechanical creep
$C_{{{\text{MB}}}}^{{\prime }}$: Compression ratio coupling mechanical creep and bio-compression
$C_{{{\text{RS}}}}^{{\prime }}$: Residual compression ratio
*H*: Waste height once the landfilling is completed
*t*_MB_: Starting date of waste settlement by mechanical creep and biodegradation (days)
*t*_RS_: Starting date of residual settlement (days)
*t*: Elapsed days (days)
