# Supplementary material for: Coupled mechanical creep and bio-compression and residual settlement in a multi-stage municipal solid waste landfill, Korea
Source: Sci Rep. 2022 Nov 9;12:19058. doi: 10.1038/s41598-022-21872-3 (PMC9646810; doi:10.1038/s41598-022-21872-3)
Supplement: Supplementary file 1 — Supplementary Information. [file 41598_2022_21872_MOESM1_ESM.docx]

**Supplementary information for:**

**Coupled mechanical creep and bio-compression and residual settlement in a multi-stage municipal solid waste landfill, Korea**

Young-Seok, Jo **^1,*^**, Wan-Kyu, Yoo **^2^**, Seong-Phil, Hwang **^2^**, Chang-Yong, Kim **^3^**

**^1,*^** Post-doctoral researcher, Korea Institute of Civil Engineering and Building Technology, Goyang, Republic of Korea (*Corresponding author*, Email: [joyoungseok@kict.re.kr](mailto:joyoungseok@kict.re.kr), Tel. +82-31-995-0971, Fax +82-31-910-0211)

**^2^** Senior researcher, Korea Institute of Civil Engineering and Building Technology, Goyang, Republic of Korea

**^3^** Senior research fellow, Korea Institute of Civil Engineering and Building Technology, Goyang, Republic of Korea

**Table S1.** The annual composition of MSW and C&D waste buried in landfill #1

| Year | MSW **^a^**  (%) | Construction and Demolition (C&D) waste | | | | | |
| --- | --- | --- | --- | --- | --- | --- | --- |
|  |  | Dredge soil | Sludge | Industrial | Construction | Textiles | Miscelllaneous |
| 1992 | 74.3 | 0.0 | 10.7 | 9.1 | 0.0 | 4.4 | 1.5 |
| 1993 | 79.5 | 0.7 | 6.5 | 3.2 | 5.9 | 2.4 | 1.8 |
| 1994 | 56.3 | 1.1 | 5.4 | 1.8 | 33.3 | 1.3 | 0.7 |
| 1995 | 58.4 | 3.2 | 8.6 | 1.9 | 26.2 | 0.7 | 1.1 |
| 1996 | 60.2 | 3.8 | 10.5 | 1.7 | 21.6 | 1.5 | 0.7 |
| 1997 | 59.9 | 3.5 | 12.3 | 0.7 | 21.9 | 1.2 | 0.4 |
| 1998 | 58.7 | 6.6 | 13.1 | 0.6 | 18.7 | 2.0 | 0.3 |
| 1999 | 56.9 | 4.7 | 10.0 | 0.5 | 25.2 | 2.4 | 0.3 |
| 2000 | 57.9 | 2.3 | 8.1 | 0.2 | 27.1 | 3.8 | 0.5 |

**^a^** The detailed MSW composition is presented in Table S3. The MSW composition in Table S3 is determined after the MSW composition in Table S1 is converted to 100 %

**Table S2.** The time history of waste disposal for each lift of waste blocks

| Lift | Waste Block | | | | | | | | | | |
| --- | --- | --- | --- | --- | --- | --- | --- | --- | --- | --- | --- |
|  | B | C | D | E | G | H | I | J | K | L | M |
| 1^st^ | 93-01-05 | 92-09-25 | 93-04-07 | 93-06-10 | 93-02-26 | 96-01-27 | 93-03-05 | 93-10-23 | 93-05-27 | 93-01-12 | 93-05-10 |
| 2^nd^ | 93-02-26 | 93-02-26 | 93-11-07 | 93-10-23 | 93-06-07 | 94-02-27 | 93-03-15 | 93-11-23 | 94-01-09 | 93-04-08 | 93-06-07 |
| 3^rd^ | 93-11-10 | 94-08-08 | 94-08-22 | 94-01-06 | 94-01-27 | 94-04-06 | 94-06-13 | 94-06-10 | 94-09-08 | 94-01-24 | 94-03-21 |
| 4^th^ | 95-10-05 | 95-02-07 | 95-06-26 | 95-09-04 | 95-01-10 | 95-01-10 | 95-01-10 | 95-02-10 | 95-08-10 | 95-05-06 | 95-05-23 |
| 5^th^ | 96-03-20 | 95-12-26 | 96-04-23 | 96-02-16 | 95-12-27 | 96-01-29 | 96-08-13 | 96-10-19 | 96-06-14 | 96-08-13 | 96-05-29 |
| 6^th^ | 97-03-13 | 96-11-28 | 97-04-10 | 97-01-28 | 96-11-25 | 98-01-22 | 97-03-18 | 97-08-22 | 97-09-09 | 97-07-10 | 97-01-29 |
| 7^th^ | 97-11-11 | 97-09-23 | 98-05-20 | 97-10-23 | 98-01-05 | 98-10-15 | 99-07-08 | 99-08-13 | 99-07-22 | 99-06-18 | 98-08-18 |
| 8^th^ | 00-05-26 | 00-04-26 | 00-05-31 | 00-04-02 | 00-03-20 | 00-04-27 | 00-07-01 | 00-09-20 | 00-09-22 | 00-08-11 | 99-12-17 |

**Table S3.** The composition and unit weight of annual MSW buried in Landfill #1 (GLC, 2000)

| Year | Waste composition (%) | | | | | | | | | | | | Unit weight  (kN/m^3^) |
| --- | --- | --- | --- | --- | --- | --- | --- | --- | --- | --- | --- | --- | --- |
|  | Organic matters | | | | | | Inorganic matters | | | | | |  |
|  | Food | Paper | Wood | Tex-tiles | R&L**^a^** | Sub-total | Plas-tics | Coal  ash | Metal | Soil | Glass | etc. |  |
| 1992 | 26.8 | 19.9 | 3.1 | NR **^b^** | 2.4 | 52.2 | 6.0 | 20.4 | 8.4 | 13.0 | NR | 0.0 | 11.07 |
| 1993 | 30.7 | 20.5 | 4.8 |  | 4.4 | 60.4 | 6.2 | 10.1 | 7.4 | 2.0 |  | 13.8 | 11.04 |
| 1994 | 39.3 | 12.0 | 4.7 |  | 4.7 | 60.7 | 6.2 | 6.1 | 2.7 | 6.0 |  | 18.1 | 11.02 |
| 1995 | 45.8 | 10.7 | 3.7 |  | 4.8 | 65.0 | 4.7 | 4.7 | 3.6 | 2.8 |  | 19.3 | 11.00 |
| 1996 | 41.2 | 17.4 | 4.5 |  | 3.6 | 66.7 | 6.0 | 2.5 | 3.6 | 2.3 |  | 18.9 | 10.98 |
| 1997 | 37.6 | 18.7 | 4.9 |  | 3.7 | 64.9 | 6.9 | 1.6 | 3.3 | 4.4 |  | 18.8 | 10.95 |
| 1998 | 35.8 | 20.4 | 4.9 |  | 3.8 | 64.9 | 6.9 | 1.6 | 3.3 | 2.9 |  | 20.5 | 10.93 |
| 1999 | 32.1 | 28.7 | 1.6 | 4.9 | 3.6 | 70.9 | 16.5 | 0.0 | 3.3 | N/A | 4.8 | 4.5 | 10.91 |
| 2000 | 36.1 | 25.3 | 1.1 | 4.4 | 3.4 | 70.3 | 20.8 | 0.0 | 1.5 | N/A | 4.8 | 2.6 | 10.89 |

**^a^** Rubber and leather; **^b^** Not Reported;

**Table S4.** The detailed composition of organic matter for each block in Table 1

| Block | Composition (%) | | | | |
| --- | --- | --- | --- | --- | --- |
|  | Food | Paper | Wood | Textiles | Rubber & Leather |
| B | 35.6 | 17.2 | 3.5 | 4.1 | 3.7 |
| C | 34.9 | 16.8 | 3.6 | 4.2 | 3.7 |
| D | 35.7 | 16.7 | 3.8 | 4.1 | 3.8 |
| E | 36.1 | 16.4 | 3.7 | 4.1 | 3.8 |
| G | 35.8 | 16.6 | 3.7 | 4.1 | 3.9 |
| H | 36.9 | 16.0 | 3.5 | 4.1 | 3.8 |
| I | 35.4 | 17.9 | 3.4 | 4.4 | 3.8 |
| J | 35.4 | 17.9 | 3.4 | 4.4 | 3.8 |
| K | 35.4 | 18.0 | 3.4 | 4.4 | 3.8 |
| L | 35.4 | 17.9 | 3.4 | 4.4 | 3.8 |
| M | 34.9 | 17.1 | 3.9 | 4.6 | 3.8 |

**Reference**

GLC. Final Design Report for Landfill Stabilization of Landfill #1. Incheon, Korea: Gimpo Metropolitan Landfill Corporation (2000).
